# Severe pediatric adenoviral pneumonia combined with invasive pulmonary aspergillosis

**DOI:** 10.1186/s12890-023-02447-y

**Published:** 2023-05-01

**Authors:** Shuihua Huang, Shengxin Zhang, Lin Yuan, Zhiqiang Zhuo, Xingdong Wu

**Affiliations:** 1 Department of Infection, Xiamen Children’s Hospital, Xiamen Branch of Fudan University Pediatric Hospital, Xiamen, Fujian China; 2 Pediatric intensive care unit, Xiamen Children’s Hospital, Xiamen Branch of Fudan University Pediatric Hospital, Xiamen, Fujian China

**Keywords:** Adenovirus, Invasive pulmonary aspergillosis, Pneumonia, Bronchoscopy, Pediatric

## Abstract

**Background:**

This study aimed to analyze the clinical characteristics of severe pediatric adenoviral pneumonia combined with invasive pulmonary aspergillosis.

**Methods:**

We retrospectively analyzed the clinical data of five children clinically diagnosed with severe adenoviral pneumonia combined with invasive pulmonary aspergillosis at Xiamen Children’s Hospital.

**Results:**

These five children included one boy and four girls, with ages of onset ranging from 8 months and 15 days to 2 years and 2 months. All of them had fever with a mean duration of 11–35 days and cough. Pulmonary imaging was performed, which revealed solid pulmonary opacification in all five children, pleural effusion in two children, and emphysema and multiple small cavity formations in one child. Multiple microbiological tests were performed on the 5 children, and adenovirus was positive in the alveolar lavage fluid for the first time, and aspergillus culture was positive in the second test. On tracheoscopy, the bronchial mucosa was seen to be congested and edematous or pale and eroded; white moss-like material was seen adhering to the tracheal wall or even blocking the airway. The five children were treated with a combination of two or more broad-spectrum antimicrobials, glucocorticoids, and gamma globulins and underwent bronchoscopy. Voriconazole was added in the treatment regimen after the diagnosis of aspergillosis (28–34 days of treatment). Four of the children were discharged in good condition with a mean total length of hospital stay of 17–47 days. The other child leave against medical advice. Follow-up 3–5 months after discharge showed that one child had been cured; two children had developed obliterative bronchiolitis; one child had developed bronchiectasis; and the remaining child who had been discharged spontaneously was not contactable via telephone.

**Conclusions:**

Immune disorders and antibiotic and steroid treatments for adenovirus infection are high-risk factors for secondary invasive pulmonary aspergillosis in children. Prolonged fever and cough are the main manifestations, but which lack specificity, and bronchoscopic mucosal-specific injury evaluation and alveolar lavage fluid culture are helpful in the diagnosis of aspergillosis. The long-term prognosis of severe pediatric adenoviral pneumonia combined with invasive pulmonary aspergillosis maybe poor.

## Introduction

Adenoviral (ADV) pneumonia is one of the most serious types of community-acquired pneumonia in children [1–3]. It has received attention from physicians at all levels because of its severe clinical presentation, susceptibility to multisystem organ complications, and lack of specificity in treatment. Active and effective treatment has reduced its mortality rate and long-term complications in children to some extent. In recent years, we have found some cases of severe ADV pneumonia complicated with fungal infections, especially aspergillus. Treatment is more difficult because of co-infection. Aspergillus is a conditionally pathogenic organism widely present in the natural environment, and lung tissue is the most common target organ for aspergillosis. If there is pulmonary involvement, it is referred to as invasive pulmonary aspergillosis (IPA) [4]. Aspergillosis often presents insidiously and lacks clinical specificity[5]. Aspergillosis secondary to ADV infection is even more difficult to detect, and without aggressive treatment, it will have a poor prognosis or even cause death; thus, it should not be taken lightly by physicians at all levels. We have diagnosed five children with severe ADV pneumonia combined with IPA since 2016. In this study, we reviewed and analyzed their clinical characteristics to provide a basis for early clinical diagnosis and treatment.

## Materials and methods

### General clinical information

Five children clinically diagnosed with ADV pneumonia combined with IPA at Xiamen Children’s Hospital since 2016 were evaluated. They included one boy and four girls, with age of onset ranging from 8 months and 15 days to 2 years and 2 months. Therefore, all of them were under 3 years of age, including one child under 1 year of age. One patient had a history of severe pneumonia treated with antibiotics and methylprednisolone 1 month prior to admission, and the other four patients had previously been healthy.

### Diagnostic criteria

For the diagnostic criteria for ADV pneumonia, we referred to Zhu Futang Practical Pediatrics [6]. For the diagnostic criteria for severe pneumonia, we referred to the Code of Practice for the Treatment of Community-Acquired Pneumonia in Children (2019 edition) [7]: (i) poor general condition; (ii) impaired consciousness; (iii) cyanosis, increased respiration (respiratory rate [RR] of ≥ 60 breaths/min at < 2 months of age; RR of ≥ 50 breaths/min at 2 months to 1 year of age; RR of ≥ 40 breaths/min at 1–5 years of age; RR of ≥ 30 breaths/min at > 5 years of age), assisted breathing (moaning, nasal flapping, or the trigeminal sign), intermittent apnea, or oxygen saturation of < 92%; (iv) hyperthermia or persistent high fever for more than 5 days; (v) signs of dehydration/refusal to eat; (vi) chest radiography or computed tomography (CT) revealing lateral lung infiltration, pneumothorax, lung necrosis, or lung abscess in ≥ 2/3; and (vii) extra-pulmonary complications. Pulmonary aspergillosis was diagnosed in accordance with the revised EORTC/MSG criteria[5]. The diagnosis was based on four components: host (high-risk) factors, clinical manifestations, microbiological evidence, and histopathological findings. It was also divided into three levels: confirmed, clinical, and proposed diagnoses. A proposed diagnosis of IPA required the presence of host factors and clinical manifestations; a clinical diagnosis required the presence of host factors, clinical manifestations, and microbiological evidence; and a confirmed diagnosis required the presence of positive lung histopathological evidence or lung tissue culture.

## Results

### Clinical manifestations

All children had high fever with temperature peaks of 39–40 °C and cough. The fever duration was ≥ 10 days in all patients, including two with a fever duration of ≥ 30 days. Two of the children had fever reappearing at 2 and 4 days, respectively, after it had improved under conventional ADV pneumonia treatment. Wet rales and sputum sounds could be heard in the lungs of all five children, and wheezing sounds could be heard in three children. The main clinical data are shown in Table 1.

**Table 1:** Basic clinical data of the five children with severe adenoviral pneumonia complicated with invasive pulmonary aspergillosis

No. and sex	Age	Symptoms	Aspergillosis duration (day)	Fever duration (day)	Symptoms on admission	Procalcitonin level (ug/L)	Albumin level (g/L)	Lactose dehydrogenase level U/L	Blood coagulation	Etiology	Chest imaging results
1 M	1Y1M	Cough, fever	30	14	Sputum sounds, stridor	2.28	29.9	589	Elevated D-dimer level	*Aspergillus fumigatus* + Adenovirus	Scattered patchy high-density shadows in both lungs, multiple small cavities in the lower lobe of the left lung, punctate in the basal segment of the left lung, emphysema
2 F	2Y2M	Cough, fever	18	16	Sputum sounds	0.23	Normal	452	Elevated D-dimer level	*A. fumigatus* + Adenovirus	Scattered patchy high-density shadows in both lungs
3 F	2Y2M	Cough, fever	14	10	Sputum sounds	0.20	29.6	2122	Elevated D-dimer level	*A. fumigatus* + Adenovirus + *Acinetobacter agrobacterium*	Scattered masses and large flakes in both lungs, high-density shadows
4 F	8M15D	Cough, fever, poor condition	38	30	Severe sputum sounds, light stridor	0.46	28.0	2194	Elevated D-dimer level, reduced fibrinogen level	*A. terreus* + Adenovirus + *Mycoplasma pneumoniae* + parainfluenza virus	Scattered patchy high-density shadows in both lungs, small pleural effusion on the left side
5 F	2Y2M	Cough, fever, poor condition, mental fatigue	58	35	Sputum sounds, stridor	0.39	34.2	718	Elevated D-dimer level, reduced fibrinogen level	*A. fumigatus* + Adenovirus	Scattered patchy high-density shadows in both lungs

### Adjunctive examinations

The peripheral blood leukocyte count, neutrophil count, and C-reactive protein level were normal in four of the children. One child had elevated peripheral blood leukocyte count and C-reactive protein level. All five children had elevated calcitoninogen levels. One child had an elevated eosinophil count, and another child had an elevated immunoglobulin E level (not checked in the other four children). Three children had elevated erythrocyte sedimentation rates (not checked in one child). Four children had decreased albumin levels. All five children showed elevated lactate dehydrogenase and D-dimer levels. Two children showed decreased fibrinogen levels. Two children showed an abnormal liver function. One child showed an abnormal cardiac enzyme profile, and another child showed a decreased immunoglobulin A level. The immune function was not checked in two children.

### Pathogenesis

Seven tests for respiratory viral antigens and mycoplasma serum antibodies, the blood G test, the blood GM test, and sputum culture were performed in all five children, and fiberoptic bronchoscopy and alveolar lavage fluid pathogenesis were completed. Multiple microbiological tests were performed on the 5 children, and adenovirus was positive in the alveolar lavage fluid for the first time, and aspergillus culture was positive in the second test. The time of diagnosis of aspergillus in 5 cases was about 5–19 days after the diagnosis of adenovirus and 6–17 days after the use of glucocorticoids. Four children were positive for *Aspergillus fumigatus* and one for *A. terreus*. Three children had positive blood G (1,3-beta-D glucan antigen test) and GM test (galactomannan antigen test) findings. One case was combined with *Agrobacterium tumefaciens* infection and another case with parainfluenza virus and mycoplasma (> 1:320).

### Imaging tests

**Fig. 1:**
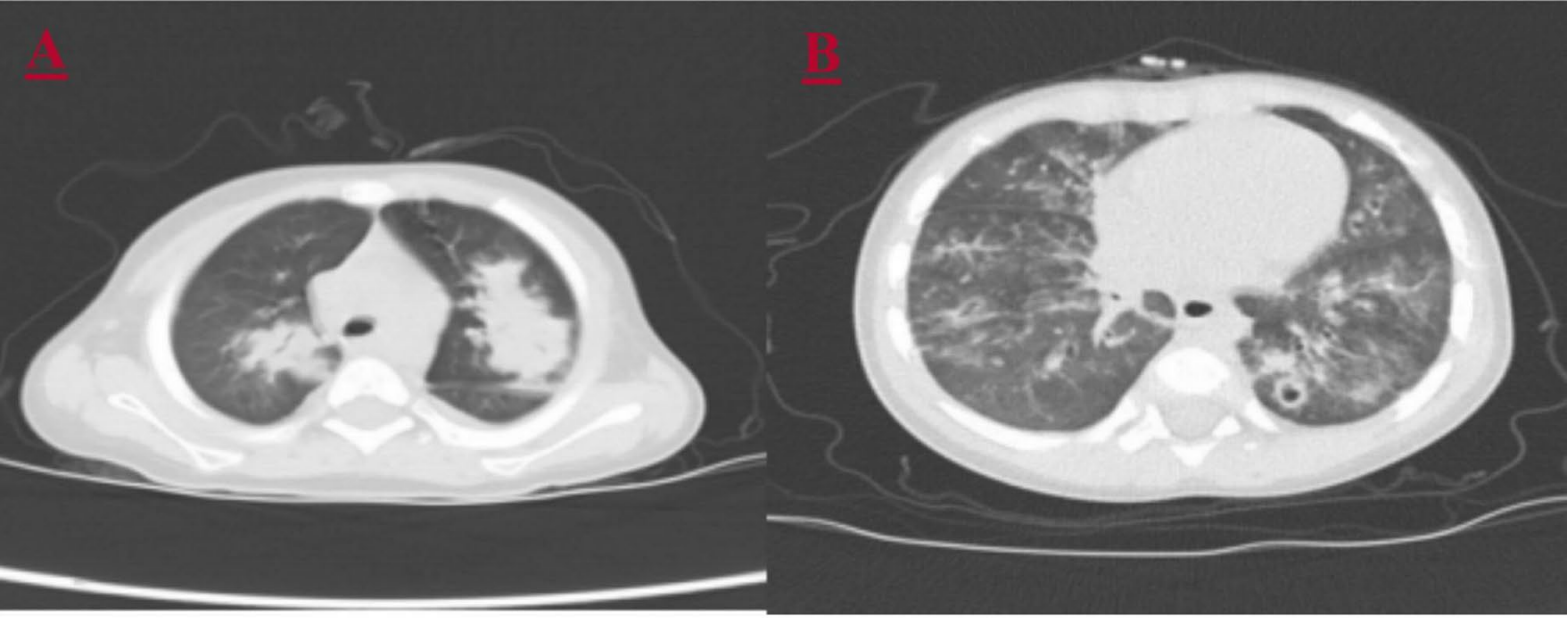
Pulmonary computed tomography changes in the children with severe adenoviral pneumonia with invasive aspergillosis: (**A**) scattered mass-like high-density shadow in both lungs; (**B**) multiple small cavity formations in the lower lobe of the left lung

All five children had solid pulmonary opacification; two had pleural effusion; and one had emphysema and multiple small cavity formations. The imaging changes are shown in Fig. 1.

### Fiberoptic bronchoscopy

**Fig. 2:**
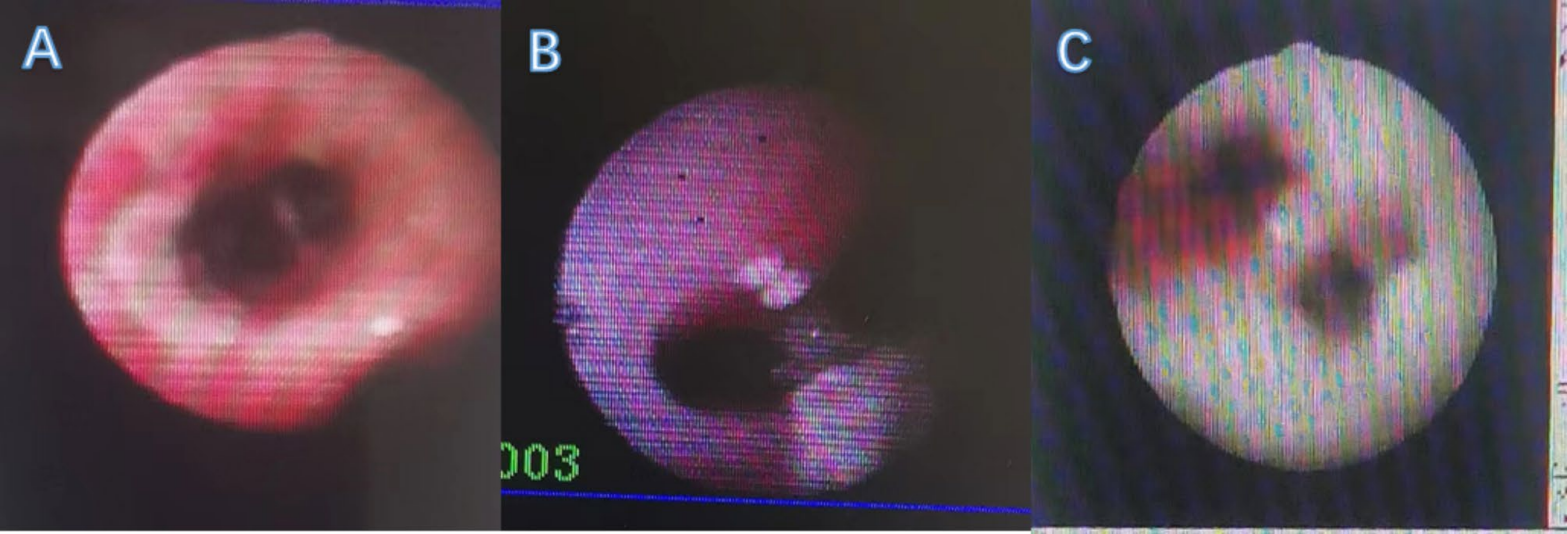
Tracheoscopic changes: (**A**) congestion and erosion of the airway mucosa; (**B**) milky plaques and pseudomembranes at the tracheal bulge; (**C**) white moss-like material widely attached to the tracheal wall

Fiberoptic bronchoscopy was performed in all five children on days 10–40 of the disease course, specifically at least two times and up to six times in one child. The sputum had a jelly-like consistency (Fig. 2).

### Treatment and regression

After admission, all five children received oxygen therapy (nasal catheter or high flow) and were treated with two or more antibiotics for 17–43 days (mean: 26 days). All of them were also treated with methylprednisolone and gamma globulin. All children underwent bronchoscopy and received a combination of an antipyretic, rehydration, and nebulized inhalation treatment. On days 7–15 of admission, three of the children still had recurrent fever; two had fever that subsided and returned, with their cough and sputum not significantly relieved. Voriconazole( 14 mg/kg.d, bid) antifungal treatment was provided for 28–34 days, after which four children improved, with their fever controlled and their coughing episodes reduced. Pulmonary imaging was reconducted before discharge, revealing varying degrees of resolution. One child(case 5) was treated with anti-infective, antifungal, and antiviral treatments. Methylprednisolone was used as an anti-inflammatory drug and gamma globulin as an immunity-regulating agent. The child still had recurrent high fever and cough, with the pulmonary imaging findings being more progressive than before.Chest CT follow-up: Multiple exudation increased in both lungs, segmental and subsegmental atelectasis increased and the scope increased. Finally, the patient leave against medical advice. Follow-up 3–5 months after discharge showed that one child had been cured; two children had developed obliterative bronchiolitis; one child had developed bronchiectasis; and one spontaneously discharged child was uncontactable.

## Discussion

The presence of aspergillosis makes the treatment of severe pediatric ADV pneumonia more difficult. There are few reports of severe ADV pneumonia combined with IPA in China or abroad, and most of them are case reports [8, 9]. In this study, we summarized the clinical data of five cases of severe ADV pneumonia combined with IPA diagnosed in our hospital and investigated the possible pathogenesis and treatment strategies.

All five children in this study had an essentially normal routine blood leukocyte count on admission. Four of them were previously healthy and free of underlying diseases, such as primary immunodeficiency disorders; however, the etiology of IPA secondary to severe ADV pneumonia deserves to be explored. First, the use of broad-spectrum antibiotics and glucocorticoids is a high-risk factor for the development of IPA. All of the children in this study had a long history of using two or more broad-spectrum antimicrobials and glucocorticoids, and some of them had also undergone frequent invasive surgeries and/or received multiple tube placements, which significantly increased the incidence of invasive fungal infections. Second, ADV infection causes airway mucosal congestion, edema, necrosis, and even shedding or destruction, which consequently reduces the airway’s ability to clear pathogenic bacteria; the shedding of necrotic material also tends to block the airway lumen, thereby increasing the risk of fungal infections. Rong et al. reported that the formation of cavities in the lungs after the development of ADV infection combined with IPA was closely related to the removal of necrotic shedding of lung tissue [9]. Furthermore, ADV infection stimulates the secretion of a large number of immune cells causing a storm of inflammatory factors and damage to lung tissue cells and pulmonary vascular endothelial cells causing pulmonary atelectasis and ARDS, greatly increasing the possibility of secondary infection by other pathogens (including aspergillus). The literature shows that ADV infection induces the release of inflammatory cytokines, such as IL-1, IL-6, and tumor necrosis factor-α, which stimulate immune cells, such as natural killer cells and macrophages, to migrate to the lungs, thereby exacerbating lung injury[10, 11].

The clinical presentation of IPA lacks specificity, and there is no exception to this tendency in severe ADV infection combined with IPA. The five children in this study mainly presented with fever and cough, wet rales, sputum, and wheezing sounds, and these signs and symptoms are of minimal help in guiding pathogenic differentiation. The only guiding factor is that fever and cough in children that do not improve for a long time under standard treatment or reappear after improvement are a warning for fungal (including aspergillus) infections. Hemorrhagic pulmonary infarction or ruptured fungal aneurysm resulting from aspergillus invasion of the vessel wall has been shown to present with hemoptysis and chest pain, which is suggestive of IPA[12]. This was not the case in the patients in this study. A subpleural wedge-shaped solid shadow, the halo sign, and the air crescent sign on pulmonary CT are considered to be specific for IPA [13]; however, none of the five children in this study had these characteristic changes, suggesting that pulmonary CT also has limitations in the diagnosis of IPA, and the absence of characteristic pulmonary CT findings cannot exclude it.

We believe that both bronchoscopic techniques and alveolar lavage fluid culture have positive implications for the treatment of aspergillosis (especially severe ADV infection complicated by IPA). With the gradual development of bronchoscopic techniques in children, the range of indications has been gradually expanded, especially for diseases, such as severe pneumonia [14]. In this study, all five children underwent bronchoscopies, and some of them even underwent them several times; the congested sputum, sputum clots, and white moss-like adhesions in their lungs were removed, which effectively relieved the disease and shortened its course. One child in this study underwent bronchoplasty, and bronchoscopic aspiration or clamping out of the plasticized material became the primary treatment. Aspergillosis was highly suspected when a child presented with pale and vesicular tracheal walls, white moss-like attachments, and even obstruction of the airway under tracheoscopy, and the subsequent positive pathogenic culture results for aspergillus verified the initial judgment; thus, the abovementioned changes under tracheoscopy were considered to be a strong indication of complicated IPA. Similar tracheoscopic manifestations have easily been observed by scholars both nationally and internationally, suggesting the same clinical inference [15, 16]. In our study, the alveolar lavage fluid cultures of all five children yielded positive findings for ADV and aspergillus, with a diagnostic positivity rate of 100%; this rate was significantly higher than that of the other tests, such as the blood and sputum cultures and GM tests, which played a decisive role in the definitive diagnosis in this study. There are reports both in China and internationally showing satisfactory results after application of bronchoscopy and alveolar lavage fluid testing by medical personnel in clinical settings[17]. Hence, the 2016 Infectious Disease Society of America guidelines for aspergillosis contain some recommendations for the diagnostic role of bronchoscopy for this disease [13]. Notably, even though the positivity rate of aspergillus in alveolar lavage fluid cultures is high, culture findings generally take approximately 3 days to obtain; thus, there are limitations in terms of timeliness. Currently, some scholars use alveolar lavage fluid for GM tests. The positivity rate is similar to that of culture, but with substantially better timeliness[18, 19], suggesting that the GM test can be used in alveolar lavage fluid in the future — a topic for further exploration.

Severe ADV pneumonia is difficult to treat, with the literature showing that the mortality rate can be as high as 10% and that 14–60% of those who survive may have varying degrees of residual disease [20]. Prognosis is further affected when other comorbidities (e.g., secondary fungal infections, including aspergillus) are present. In this study, four of the five children diagnosed with IPA were discharged with significant improvements in the symptoms after antifungal treatment with voriconazole, and one child was discharged from treatment. Follow-up 3–5 months after discharge showed recovery in one child, obliterative bronchiolitis in two children, bronchiectasis in one child, and unsuccessful contact attempts in one child who leave against medical advice. The proportion of their long-term sequelae was at least 60%, suggesting a more dismal prognosis for severe ADV pneumonia combined with aspergillosis.

In conclusion, severe pediatric ADV pneumonia combined with IPA is not common. Immune disorders caused by ADV infection and antimicrobial and hormonal applications are possible causes of secondary IPA. Prolonged fever and cough are the main manifestations, but which lack specificity. Bronchoscopic mucosal-specific damage evaluation and alveolar lavage fluid culture are helpful in the diagnosis of aspergillosis, and the disease is prone to causing obliterative bronchiolitis and other diseases. The prognosis maybe poor because of the possible complications.

## Data Availability

All data generated or analysed during this study are included in this published article. The raw data supporting the conclusions of this article will be made available by the authors.
